# Sharp Congruences Adequate with Temporal Logics Combining Weak and Strong Modalities

**DOI:** 10.1007/978-3-030-45237-7_4

**Published:** 2020-03-13

**Authors:** Frédéric Lang, Radu Mateescu, Franco Mazzanti

**Affiliations:** 8 Johannes Kepler University, Linz, Austria; 9 University of Birmingham, Birmingham, UK; 10 Univ. Grenoble Alpes, Inria, CNRS, Grenoble INP, LIG, 38000 Grenoble, France; 11 ISTI-CNR, Pisa, Italy

**Keywords:** Bisimulation, Concurrency, Model checking, Mu-calculus

## Abstract

We showed in a recent paper that, when verifying a modal $\mu$-calculus formula, the actions of the system under verification can be partitioned into sets of so-called weak and strong actions, depending on the combination of weak and strong modalities occurring in the formula. In a compositional verification setting, where the system consists of processes executing in parallel, this partition allows us to decide whether each individual process can be minimized for either divergence-preserving branching (if the process contains only weak actions) or strong (otherwise) bisimilarity, while preserving the truth value of the formula. In this paper, we refine this idea by devising a family of bisimilarity relations, named sharp bisimilarities, parameterized by the set of strong actions. We show that these relations have all the nice properties necessary to be used for compositional verification, in particular congruence and adequacy with the logic. We also illustrate their practical utility on several examples and case-studies, and report about our success in the RERS 2019 model checking challenge.

## 1 Introduction

This paper deals with the verification of action-based, branching-time temporal properties expressible in the modal $\mu$-calculus ($L_\mu$) [31] on concurrent systems consisting of processes composed in parallel, usually described in languages with process algebraic flavour. A well-known problem is the state-space explosion that happens when the system state space exceeds the available computer memory.

Compositional verification is a set of techniques and tools that have proven efficient to palliate state-space explosion in many case studies [18]. They may either focus on the construction of the state space reduced for some equivalence relation, such as compositional state space construction [24, 32, 36, 43, 45–47], or on the decomposition of the full system verification into the verification of (expectedly smaller) subsystems, such as compositional reachability analysis [49, 10], assume-guarantee reasoning [41], or partial model checking [1, 34].

** Institute of Engineering Univ. Grenoble Alpes

In this paper, we focus on property-dependent compositional state space construction, where the reduction to be applied to the system is obtained by analysing the property under verification. We will refine the approach of [37] which, given a formula $\varphi$ of $L_\mu$ to be verified, shows how to extract from $\varphi$ a maximal hiding set of actions and a reduction (minimization for either strong [40] or divergence-preserving[3] branching — divbranching for short — bisimilarity [20, 23]) that preserves the truth value of $\varphi$. The reduction is chosen according to whether $\varphi$ belongs to an $L_\mu$ fragment named $L_\mu^{dbr}$, which is adequate with divbranching bisimilarity. This fragment consists of $L_\mu$ restricted to *weak* modalities, which match actions preceded by (property-preserving) sequences of hidden actions, as opposed to traditional strong modalities $\langle\alpha\rangle\,\varphi_0$ and $[\alpha]\,\varphi_0$, which match only a single action satisfying $\alpha$. If $\varphi$ belongs to $L_\mu^{dbr}$, then the system can be reduced for divbranching bisimilarity; otherwise, it can be reduced for strong bisimilarity, the weakest congruence preserving full $L_\mu$. We call this approach of [37] the mono-bisimulation approach.

We refine the mono-bisimulation approach in [35], by handling the case of $L_\mu$ formulas containing both strong and weak modalities. To do so, fragments named $L_\mu^{strong}(A_s)$ extend $L_\mu^{dbr}$ with strong modalities matching only the actions belonging to a given set $A_s$ of *strong* actions. This induces a partition of the parallel processes into those containing at least one strong action and those not containing any, so that a formula $\varphi \in L_\mu^{strong}(A_s)$ is still preserved if the processes containing strong actions are reduced for strong bisimilarity and the other ones for divbranching bisimilarity. We call this refined approach the combined bisimulations approach. Guidelines are also provided in [35] to extract a set of strong actions from particular $L_\mu$ formulas encoding the operators of widely-used temporal logics, such as CTL [11], ACTL [39], PDL [15], and PDL-$\Delta$ [44]. This approach is implemented on top of the CADP verification toolbox [19], and experiments show that it can improve the capabilities of compositional verification on realistic case studies, possibly reducing state spaces by orders of magnitude.

In this paper, we extend these results as follows: (1) We refine the approach by devising a family of new bisimilarity relations, called *sharp bisimilarities*, parameterized by the set of strong actions $A_s$. They are hybrid between strong and divbranching bisimilarities, where strong actions are handled as in strong bisimilarity whereas weak actions are handled as in divbranching bisimilarity. (2) We show that each fragment $L_\mu^{strong}(A_s)$ is adequate with the corresponding sharp bisimilarity, namely, $L_\mu^{strong}(A_s)$ is precisely the set of properties that are preserved by sharp bisimilarity (w.r.t. $A_s$) on all systems. (3) We show that, similarly to strong and divbranching bisimilarities, every sharp bisimilarity is a congruence for parallel composition, which enables it to be used soundly in a compositional verification setting. (4) We define an efficient state space

[3] In [18, 37], the name *divergence-sensitive* is used instead of *divergence-preserving* branching bisimulation (or branching bisimulation with explicit divergences) [20, 23]. This could lead to a confusion with the relation defined in [13], also called *divergence-sensitive* but slightly different from the former relation. To be consistent in notations, we replace by *dbr* the abbreviation *dsbr* used in earlier work.

reduction algorithm that preserves sharp bisimilarity and has the same worst-case complexity as divbranching minimization. Although it is not a minimization (i.e., sharp bisimilar states may remain distinguished in the reduced state space), it coincides with divbranching minimization whenever the process it is applied to does not contain strong actions, and with strong minimization in the worst case. Therefore, applying this reduction compositionally always yields state space reduction at least as good as [35], which itself is an improvement over [37]. (5) At last, we illustrate our approach on case studies and compare our new results with those of [35, 37]. We also report about our recent success in the RERS 2019 challenge, which was obtained thanks to this new approach.

The paper is organized as follows: Sections 2 and 3 introduce the necessary background about process descriptions and temporal logic. Section 4 defines sharp bisimilarity, states its adequacy with $L_\mu^{strong}(A_s)$, and its congruence property for parallel composition. Section 5 presents the reduction algorithm and shows that it is correct and efficient. Section 6 illustrates our new approach on the case studies. Section 7 discusses related work. Finally, Section 8 concludes and discusses research directions for the future. The proofs of all theorems presented in this paper and a detailed description of how we tackled the RERS 2019 challenge are available in a Zenodo archive.[4]

## 2 Processes, Compositions, and Reductions

We consider systems of processes whose behavioural semantics can be represented using an LTS (*Labelled Transition System*).

**Definition 1 (LTS).** *Let $\mathcal{A}$ be an infinite set of actions including the invisible action $\tau$ and visible actions $\mathcal{A} \setminus \{\tau\}$. An LTS $P$ is a tuple $(\Sigma, A, \longrightarrow, p_{init})$, where $\Sigma$ is a set of states, $A \subseteq \mathcal{A}$ is a set of actions, $\longrightarrow \subseteq \Sigma \times A \times \Sigma$ is the (labelled) transition relation, and $p_{init} \in \Sigma$ is the initial state. We may write $\Sigma_P, A_P, \longrightarrow_P$ for the sets of states, actions, and transitions of an LTS $P$, and $init(P)$ for its initial state. We assume that $P$ is finite and write $|P|_{st}$ (resp. $|P|_{tr}$) for the number of states (resp. transitions) of $P$. We write $p \xrightarrow{a} p'$ for $(p, a, p') \in \longrightarrow$ and $p \xrightarrow{A}$ for $(\exists p' \in \Sigma_P, a \in A)\ p \xrightarrow{a} p'$.*

LTS can be composed in parallel and their actions may be abstracted away using the parallel composition and action mapping defined below, of which action hiding, cut (also known as restriction), and renaming are particular cases.

**Definition 2 (Parallel composition of LTS).** *Let $P, Q$ be LTS and $A_{sync} \subseteq \mathcal{A} \setminus \{\tau\}$. The parallel composition of $P$ and $Q$ with synchronization on $A_{sync}$, written "$P\ |[A_{sync}]|\ Q$", is defined as $(\Sigma_P \times \Sigma_Q, A_P \cup A_Q, \longrightarrow, (init(P), init(Q)))$, where $(p, q) \xrightarrow{a} (p', q')$ if and only if (1) $p \xrightarrow{a}_P p'$, $q' = q$, and $a \notin A_{sync}$, or (2) $p' = p$, $q \xrightarrow{a}_Q q'$, and $a \notin A_{sync}$, or (3) $p \xrightarrow{a}_P p'$, $q \xrightarrow{a}_Q q'$, and $a \in A_{sync}$.*

[4] https://doi.org/10.5281/zenodo.3470930

**Definition 3 (Action mapping).** *Let $P$ be an LTS and $\rho : A_P \to 2^{\mathcal{A}}$ be a total function. We write $\rho(A_P)$ for the image of $\rho$, defined by $\bigcup_{a \in A_P} \rho(a)$. We write $\rho(P)$ for the LTS $(\Sigma_P, \rho(A_P), \longrightarrow, init(P))$ where $\longrightarrow = \{(p, a', p') \mid (\exists a \in A_P)\ p \xrightarrow{a}_P p' \wedge a' \in \rho(a)\}$. An action mapping $\rho$ is admissible if $\tau \in A_P$ implies $\rho(\tau) = \{\tau\}$. We distinguish the following admissible action mappings:*

- *$\rho$ is an action hiding if $(\exists A \subseteq \mathcal{A} \setminus \{\tau\})\ (\forall a \in A \cap A_P)\ \rho(a) = \{\tau\} \wedge (\forall a \in A_P \setminus A)\ \rho(a) = \{a\}$. We write "**hide** $A$ **in** $P$" for $\rho(P)$.*
- *$\rho$ is an action cut if $(\exists A \subseteq \mathcal{A} \setminus \{\tau\})\ (\forall a \in A \cap A_P)\ \rho(a) = \emptyset \wedge (\forall a \in A_P \setminus A)\ \rho(a) = \{a\}$. We write "**cut** $A$ **in** $P$" for $\rho(P)$.*
- *$\rho$ is an action renaming if $(\exists f : A_P \to \mathcal{A})\ (\forall a \in A_P)\ \rho(a) = \{f(a)\}$ and $\tau \in A_P$ implies $f(\tau) = \tau$. We write "**rename** $f$ **in** $P$" for $\rho(P)$.*

Parallel composition and action mapping subsume all abstraction and composition operators encodable as *networks of LTS* [42, 18, 33], such as synchronization vectors[5] and the parallel composition, hiding, renaming, and cut operators of CCS [38], CSP [8], mCRL [26], LOTOS [29], E-LOTOS [30], and LNT [9].

LTS can be compared and reduced modulo well-known bisimilarity relations, such as strong [40] and (div)branching [20, 23] bisimilarity. We do not give their definitions, which can easily be found elsewhere (e.g., [35]). They are special cases of Definition 7 (page 7), as shown by Theorem 1 (page 9). We write $\sim$ (resp. $\sim_{dbr}$) for the strong (resp. divbranching) bisimilarity relation between states. We write $min_{str}(P)$ (resp. $min_{dbr}(P)$) for the quotient of $P$ w.r.t. strong (resp. divbranching) bisimilarity, i.e., the LTS obtained by replacing each state by its equivalence class. The quotient is the smallest LTS of its equivalence class, thus computing the quotient is called minimization. Moreover, these bisimilarities are congruences for parallel composition and admissible action mapping. This allows reductions to be applied at any intermediate step during LTS construction, thus potentially reducing the overall cost. However, since processes may constrain each other by synchronization, composing LTS pairwise following the algebraic structure of the composition expression and applying reduction after each composition can be orders of magnitude less efficient than other strategies in terms of the largest intermediate LTS. Finding an optimal strategy is impossible, as it requires to know the size of (the reachable part of) an LTS product without actually computing the product. One generally relies on heuristics to select a subset of LTS to compose at each step of LTS construction. In this paper, we will use the *smart reduction* heuristic [12, 18], which is implemented within the SVL [17] tool of CADP [19]. This heuristic tries to find an efficient composition order by analysing the synchronization and hiding structure of the composition.

[5] For instance, the composition of $P$ and $Q$ where action $a$ of $P$ synchronizes with either $b$ or $c$ of $Q$, can be written as $\rho(P) \,|[b, c]|\, Q$, where $\rho$ maps $a$ onto $\{b, c\}$. This example illustrates the utility to map actions into sets of actions of arbitrary size.

## 3 Temporal Logics

**Definition 4 (Modal $\mu$-calculus [31]).** *The modal $\mu$-calculus ($L_\mu$) is built from action formulas $\alpha$ and state formulas $\varphi$, whose syntax and semantics w.r.t. an LTS $P = (\Sigma, A, \longrightarrow, p_{init})$ are defined as follows:*

$$
\begin{array}{llrl}
\alpha ::= & a & [\![a]\!]_A = & \{a\} \\
| & \mathsf{false} & [\![\mathsf{false}]\!]_A = & \emptyset \\
| & \alpha_1 \vee \alpha_2 & [\![\alpha_1 \vee \alpha_2]\!]_A = & [\![\alpha_1]\!]_A \cup [\![\alpha_2]\!]_A \\
| & \neg\alpha_0 & [\![\neg\alpha_0]\!]_A = & A \setminus [\![\alpha_0]\!]_A
\end{array}
$$

$$
\begin{array}{llrl}
\varphi ::= & \mathsf{false} & [\![\mathsf{false}]\!]_P\delta = & \emptyset \\
| & \varphi_1 \vee \varphi_2 & [\![\varphi_1 \vee \varphi_2]\!]_P\delta = & [\![\varphi_1]\!]_P\delta \cup [\![\varphi_2]\!]_P\delta \\
| & \neg\varphi_0 & [\![\neg\varphi_0]\!]_P\delta = & \Sigma \setminus [\![\varphi_0]\!]_P\delta \\
| & \langle\alpha\rangle\,\varphi_0 & [\![\langle\alpha\rangle\,\varphi_0]\!]_P\delta = & \{p \in \Sigma \mid \exists p \xrightarrow{a} p'.a \in [\![\alpha]\!]_A \wedge p' \in [\![\varphi_0]\!]_P\delta\ \} \\
| & X & [\![X]\!]_P\delta = & \delta(X) \\
| & \mu X.\varphi_0 & [\![\mu X.\varphi_0]\!]_P\delta = & \bigcup_{k\geq 0} {\Phi_0}^k_{P,\delta}(\emptyset)
\end{array}
$$

*where $X \in \mathcal{X}$ are propositional variables denoting sets of states, $\delta : \mathcal{X} \to 2^\Sigma$ is a context mapping propositional variables to sets of states, $[\,]$ is the empty context, $\delta[U/X]$ is the context identical to $\delta$ except for variable $X$, which is mapped to state set $U$, and the functional ${\Phi_0}_{P,\delta} : 2^\Sigma \to 2^\Sigma$ associated to the formula $\mu X.\varphi_0$ is defined as ${\Phi_0}_{P,\delta}(U) = [\![\varphi_0]\!]_P\delta[U/X]$. For closed formulas, we write $P \models \varphi$ (read $P$ satisfies $\varphi$) for $p_{init} \in [\![\varphi]\!]_P[\,]$.*

Action formulas $\alpha$ are built from actions and Boolean operators. State formulas $\varphi$ are built from Boolean operators, the possibility modality $\langle\alpha\rangle\,\varphi_0$ denoting the states with an outgoing transition labelled by an action satisfying $\alpha$ and leading to a state satisfying $\varphi_0$, and the minimal fixed point operator $\mu X.\varphi_0$ denoting the least solution of the equation $X = \varphi_0$ interpreted over $2^\Sigma$.

The usual derived operators are defined as follows: Boolean connectors $\mathsf{true} = \neg\mathsf{false}$ and $\varphi_1 \wedge \varphi_2 = \neg(\neg\varphi_1 \vee \neg\varphi_2)$; necessity modality $[\alpha]\,\varphi_0 = \neg\langle\alpha\rangle\,\neg\varphi_0$; and maximal fixed point operator $\nu X.\varphi_0 = \neg\mu X.\neg\varphi_0[\neg X/X]$, where $\varphi_0[\neg X/X]$ is the syntactic substitution of $X$ by $\neg X$ in $\varphi_0$. Syntactically, $\langle\rangle$ and $[]$ have the highest precedence, followed by $\wedge$, then $\vee$, and finally $\mu$ and $\nu$. To have a well-defined semantics, state formulas are syntactically monotonic [31], i.e., in every subformula $\mu X.\varphi_0$, all occurrences of $X$ in $\varphi_0$ fall in the scope of an even number of negations. Thus, negations can be eliminated by downward propagation. We now introduce the weak modalities of the fragment $L_\mu^{dbr}$, proposed in [37].

**Definition 5 (Modalities of $L_\mu^{dbr}$ [37]).** *We write $\alpha_\tau$ for an action formula such that $\tau \in [\![\alpha_\tau]\!]_A$ and $\alpha_a$ for an action formula such that $\tau \notin [\![\alpha_a]\!]_A$. We consider the following modalities, their $L_\mu$ semantics, and their informal semantics:*

| *modality name* | *notation* | $L_\mu$ *semantics* |
|---|---|---|
| *ultra-weak* | $\langle(\varphi_1?.\alpha_\tau)^*\rangle\,\varphi_2$ | $\mu X.\varphi_2 \vee (\varphi_1 \wedge \langle\alpha_\tau\rangle\,X)$ |
| *weak* | $\langle(\varphi_1?.\alpha_\tau)^*.\varphi_1?.\alpha_a\rangle\,\varphi_2$ | $\mu X.\varphi_1 \wedge (\langle\alpha_a\rangle\,\varphi_2 \vee \langle\alpha_\tau\rangle\,X)$ |
| *weak infinite looping* | $\langle\varphi_1?.\alpha_\tau\rangle\,@$ | $\nu X.\varphi_1 \wedge \langle\alpha_\tau\rangle\,X$ |

**Ultra-weak:** *p is source of a path whose transition labels satisfy $\alpha_\tau$, leading to a state that satisfies $\varphi_2$, while traversing only states that satisfy $\varphi_1$.*

**Weak:** *p is source of a path whose transition labels satisfy $\alpha_\tau$, leading to a state that satisfies $\varphi_1$ and $\langle\alpha_a\rangle\,\varphi_2$, while traversing only states that satisfy $\varphi_1$.*

**Weak infinite looping:** *p is source of an infinite path whose transition labels satisfy $\alpha_\tau$, while traversing only states that satisfy $\varphi_1$.*

We also consider the three dual modalities $[(\varphi_1?.\alpha_\tau)^*]\,\varphi_2 = \neg\langle(\varphi_1?.\alpha_\tau)^*\rangle\,\neg\varphi_2$, $[(\varphi_1?.\alpha_\tau)^*.\varphi_1?.\alpha_a]\,\varphi_2 = \neg\langle(\varphi_1?.\alpha_\tau)^*.\varphi_1?.\alpha_a\rangle\,\neg\varphi_2$, $[\varphi_1?.\alpha_\tau]\ \dashv\ = \neg\langle\varphi_1?.\alpha_\tau\rangle\,@$. The fragment $L_\mu^{dbr}$ adequate with divbranching bisimilarity consists of $L_\mu$ from which the modalities $\langle a\rangle\,\varphi$ and $[a]\,\varphi$ are replaced by the ultra-weak, weak, and weak infinite looping modalities defined above.

We identify fragments of $L_\mu$ parameterized by a set of strong actions $A_s$, as the set of state formulas whose action formulas contained in strong modalities satisfy only actions of $A_s$.

**Definition 6 ($L_\mu^{strong}(A_s)$ fragment of $L_\mu$ [35]).** *Let $A_s \subseteq A$ be a set of actions called strong actions and $\alpha_s$ be any action formula such that $[\![\alpha_s]\!]_A \subseteq A_s$, called a strong action formula. $L_\mu^{strong}(A_s)$ is defined as the set of formulas semantically equivalent to some formula of the following language:*

$$\begin{aligned}\varphi ::= &\ \mathsf{false} \mid \varphi_1 \vee \varphi_2 \mid \neg\varphi_0 \mid \langle\alpha_s\rangle\,\varphi_0 \mid X \mid \mu X.\varphi_0 \\ \mid &\ \langle(\varphi_1?.\alpha_\tau)^*\rangle\,\varphi_2 \mid \langle(\varphi_1?.\alpha_\tau)^*.\varphi_1?.\alpha_a\rangle\,\varphi_2 \mid \langle\varphi_1?.\alpha_\tau\rangle\,@\end{aligned}$$

*In the context of $L_\mu^{strong}(A_s)$, we call $\langle\alpha_s\rangle\,\varphi_0$ a strong modality.*[6]

In [35], we also provide guidelines for extracting a set $A_s$ from particular $L_\mu$ formulas encoding the operators of widely-used temporal logics, such as CTL [11], ACTL [39], PDL [15], and PDL-$\Delta$ [44].

*Example 1.* The PDL formula $[\mathsf{true}^*.a_1.a_2]\,\mathsf{true}$ belongs to $L_\mu^{strong}(\{a_2\})$ as it is semantically equivalent to $[(\mathsf{true}?.\mathsf{true})^*.\mathsf{true}?.a_1]\,[a_2]\,\mathsf{true}$. The CTL formula $\mathsf{EF}(\langle a_1\rangle\,\mathsf{true} \wedge \langle a_2\rangle\,\mathsf{true})$ belongs both to $L_\mu^{strong}(\{a_1\})$ as it is semantically equivalent to $\langle(\mathsf{true}?.\mathsf{true})^*\rangle\,\langle(\langle a_1\rangle\,\mathsf{true}?.\mathsf{true})^*.\langle a_1\rangle\,\mathsf{true}?.a_2\rangle\,\mathsf{true}$ and to $L_\mu^{strong}(\{a_2\})$ as it is semantically equivalent to the same formula where $a_1$ and $a_2$ are swapped. These formulas do not belong to $L_\mu^{strong}(\emptyset)$. (This was shown in [35].)

The latter example shows that to a formula $\varphi$ may correspond several minimal sets of strong actions $A_s$. Indeed, either the $\langle a_1\rangle\,\mathsf{true}$ or the $\langle a_2\rangle\,\mathsf{true}$ modality can be made part of a weak modality, but not both in the same formula.

## 4 Sharp Bisimilarity

We define the family of sharp bisimilarity relations below. Each relation is hybrid between strong and divbranching bisimilarities, parameterized by the set of strong actions, such that the conditions of strong bisimilarity apply to strong actions and the conditions of divbranching bisimilarity apply to all other actions.

[6] For generality we allow $\tau \in A_s$, to enable strong modalities of the form $\langle\alpha_\tau\rangle\,\varphi_0$.

**Definition 7 (Sharp bisimilarity).** *A divergence-unpreserving sharp bisimulation w.r.t. a set of actions $A_s$ is a symmetric relation $R \subseteq \Sigma \times \Sigma$ such that if $(p, q) \in R$ then for all $p \xrightarrow{a} p'$, there exists $q'$ such that $(p', q') \in R$ and either of the following hold: (1) $q \xrightarrow{a} q'$, or (2) $a = \tau$, $\tau \notin A_s$, and $q' = q$, or (3) $a \notin A_s$, and there exists a sequence of transitions $q_0 \xrightarrow{\tau} \ldots \xrightarrow{\tau} q_n \xrightarrow{a} q'$ $(n \geq 0)$ such that $q_0 = q$, and for all $i \in 1..n$, $(p, q_i) \in R$.*[7] *A sharp bisimulation $R$ additionally satisfies the following divergence-preservation condition: for all $(p_0, q_0) \in R$ such that $p_0 \xrightarrow{\tau} p_1 \xrightarrow{\tau} p_2 \xrightarrow{\tau} \ldots$ with $(p_i, q_0) \in R$ for all $i \geq 0$, there is also an infinite sequence $q_0 \xrightarrow{\tau} q_1 \xrightarrow{\tau} q_2 \xrightarrow{\tau} \ldots$ such that $(p_i, q_j) \in R$ for all $i, j \geq 0$. Two states $p$ and $q$ are sharp bisimilar w.r.t. $A_s$, written $p \sim_{\sharp A_s} q$, if and only if there exists a sharp bisimulation $R$ w.r.t. $A_s$ such that $(p, q) \in R$.*

Similarly to strong, branching, and divbranching bisimilarities, sharp bisimilarity is an equivalence relation as it is the union of all sharp bisimulations. The quotient of an LTS $P$ w.r.t. sharp bisimilarity is unique and minimal both in number of states and number of transitions.

*Example 2.* Let $a, b, \omega \in \mathcal{A} \setminus \{\tau\}$, $\tau, \omega \notin A_s$. LTS $P_i$ and $P'_i$ of Figure 1 satisfy $P_i \sim_{\sharp A_s} P'_i$ $(i \in 1..7)$. We give the smallest relation between $P_i$ and $P'_i$, whose symmetric closure is a sharp bisimulation w.r.t. $A_s$ and the weakest condition for $P'_i$ to be minimal. Unlike divbranching, states on the same $\tau$-cycle are not necessarily sharp bisimilar: in $P'_7$, if $a \in A_s$ then $p'_0$ and $p'_2$ are not sharp bisimilar.

*Example 3.* The LTS of Figure 2(a) is equivalent for $\sim_{\sharp\{a\}}$ to the one of Figure 2(b), which is minimal. We see that sharp bisimilarity reduces more than strong bisimilarity when at least one action (visible or invisible) is weak. Here, $\tau$ is the only weak action and the minimized LTS is smaller than the one minimal for strong bisimilarity (only $p_1$ and $p_2$ are strongly bisimilar).

If $\tau \in A_s$, then case (2) of Definition 7 cannot apply, i.e., $\tau$-transitions cannot be totally suppressed. As a consequence, looking at case (3), if $\tau$-transitions are present in state $q_0$ then, due to symmetry, they must have a counterpart in state $p$. As a result, finite sequences of $\tau$-transitions are preserved. Sharp may however differ from strong bisimilarity in the possibility to compress circuits of $\tau$-transitions that would remain unreduced, as illustrated in Example 4 below.

*Example 4.* If $\tau \in A_s$ and $a \notin A_s$, then the LTS of Figure 2(b) (which is minimal for strong bisimilarity) can be reduced to the LTS of Figure 2(c).

Next theorems are new. Theorem 1 expresses that sharp bisimilarity w.r.t. a set of strong actions $A_s$ is strictly stronger than w.r.t. any set of strong actions strictly included in $A_s$. Unsurprisingly, it also establishes that sharp coincides with divbranching when the set of strong actions is empty, and with strong when

[7] We require that $(p, q_i) \in R$ for all $i \in 1..n$ and not the simpler condition $(p, q_n) \in R$ (as usual when defining branching bisimulation) because sharp bisimulation has not the nice property that $(p, q_0) \in R$ and $(p, q_n) \in R$ imply $(p, q_i) \in R$ for all $i \in 1..n$.

$a \neq \tau \wedge b \neq \tau \wedge \omega \neq \tau \wedge \tau \notin A_s \wedge \omega \notin A_s$ implies

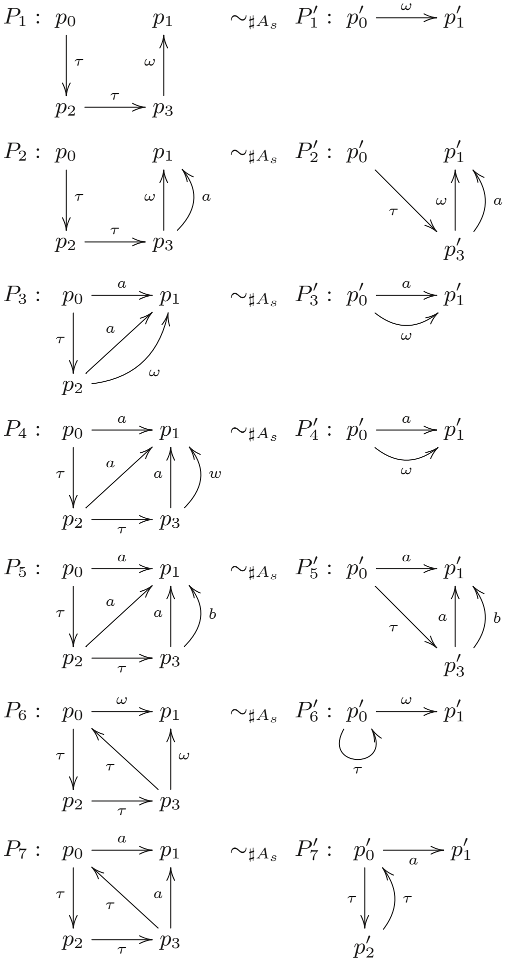

$\{(p_0, p'_0), (p_1, p'_1), (p_2, p'_0), (p_3, p'_0)\}$
$P'_1$ minimal

$\{(p_0, p'_0), (p_1, p'_1), (p_2, p'_0), (p_3, p'_3)\}$
$a \in A_s$ implies $P'_2$ minimal

$\{(p_0, p'_0), (p_1, p'_1), (p_2, p'_0)\}$
$a \neq \omega$ implies $P'_3$ minimal

$\{(p_0, p'_0), (p_1, p'_1), (p_2, p'_0), (p_3, p'_0)\}$
$a \neq \omega$ implies $P'_4$ minimal

$\{(p_0, p'_0), (p_1, p'_1), (p_2, p'_0), (p_3, p'_3)\}$
$b \neq a \wedge b \in A_s$ implies $P'_5$ minimal

$\{(p_0, p'_0), (p_1, p'_1), (p_2, p'_0), (p_3, p'_0)\}$
$P'_6$ minimal

$\{(p_0, p'_0), (p_1, p'_1), (p_2, p'_2), (p_3, p'_0)\}$
$a \in A_s$ implies $P'_7$ minimal

**Fig. 1.** Examples of sharp bisimilar LTS

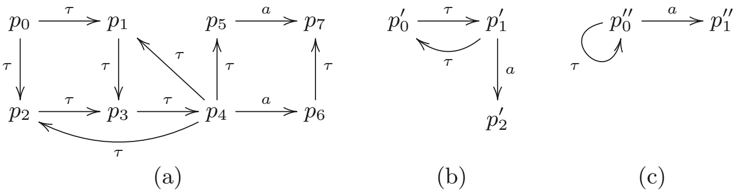

**Fig. 2.** LTS of Examples 3 and 4

it comprises all actions (including $\tau$). It follows that the set of sharp bisimilarity relations equipped with set inclusion forms a complete lattice whose supremum is divbranching bisimilarity and whose infimum is strong bisimilarity.

**Theorem 1.** *(1)* $\sim_{\sharp\emptyset} = \sim_{dbr}$ *(2)* $\sim_{\sharp\mathcal{A}} = \sim$ *(3) if* $A'_s \subset A_s$ *then* $\sim_{\sharp A_s} \subset \sim_{\sharp A'_s}$.

Theorem 2 expresses that sharp bisimilarity w.r.t. $A_s$ preserves the truth value of all formulas of $L_\mu^{strong}(A_s)$, and Theorem 3 that two LTS verifying exactly the same formulas of $L_\mu^{strong}(A_s)$ are sharp bisimilar. We can then deduce that $L_\mu^{strong}(A_s)$ is adequate with $\sim_{\sharp A_s}$, as expressed by Corollary 1.

**Theorem 2.** *If* $P \sim_{\sharp A_s} P'$ *and* $\varphi \in L_\mu^{strong}(A_s)$ *then* $P \models \varphi$ *iff* $P' \models \varphi$.

**Theorem 3.** *If* $(\forall \varphi \in L_\mu^{strong}(A_s))$ $P \models \varphi$ *iff* $Q \models \varphi$*, then* $P \sim_{\sharp A_s} Q$.

**Corollary 1.** $L_\mu^{strong}(A_s)$ *is adequate with* $\sim_{\sharp A_s}$*, i.e.,* $P \sim_{\sharp A_s} P'$ *if and only if* $(\forall \varphi \in L_\mu^{strong}(A_s))$ $P \models \varphi$ *iff* $P' \models \varphi$.

Theorems 4 and 5 express that sharp bisimilarity is a congruence for parallel composition and admissible action mapping. It follows that it is also a congruence for hide, cut, and rename, as expressed by Corollary 2.

**Theorem 4.** *If* $P \sim_{\sharp A_s} P'$*,* $Q \sim_{\sharp A_s} Q'$ *then* $P \,|[A_{sync}]|\, Q \sim_{\sharp A_s} P' \,|[A_{sync}]|\, Q'$.

**Theorem 5.** *If* $\rho$ *is admissible and* $P \sim_{\sharp A_s} P'$*, then* $\rho(P) \sim_{\sharp A'_s} \rho(P')$*, where* $A'_s = \rho(A_s) \setminus \rho(A_P \setminus A_s)$.

**Corollary 2.** *We write* $A_\tau$ *for* $A \cup \{\tau\}$*. If* $P \sim_{\sharp A_s} P'$ *then:*

– ***cut*** $A$ ***in*** $P \sim_{\sharp A_s}$ ***cut*** $A$ ***in*** $P'$
– ***hide*** $A$ ***in*** $P \sim_{\sharp A_s}$ ***hide*** $A$ ***in*** $P'$ *if* $A_\tau \subseteq A_s \vee A_\tau \cap A_s = \emptyset$
– ***rename*** $f$ ***in*** $P \sim_{\sharp A_s}$ ***rename*** $f$ ***in*** $P'$ *if* $f(A_s) \subseteq A_s \wedge f(A_P \setminus A_s) \cap A_s = \emptyset$

These theorems and corollaries generalize results on strong and divbranching bisimilarity. In particular, the side conditions of Corollary 2 are always true when $A_s = \emptyset$ (divbranching) or $A_s = \mathcal{A}$ (strong).

Since every admissible network of LTS can be translated into an equivalent composition expression consisting of parallel compositions and admissible action mappings, Theorems 4 and 5 imply some congruence property at the level of networks of LTS. However, one must be careful on how the synchronization rules preserve or modify the set of strong actions of components.

In the sequel, we establish formally the relationship between sharp bisimilarity and sharp $\tau$-confluence, a strong form of $\tau$-confluence [27] defined below in a way analogous to strong $\tau$-confluence in [28]. It is known that every $\tau$-transition that is $\tau$-confluent is inert for branching bisimilarity, i.e., its source and target states are branching bisimilar. There are situations where $\tau$-confluence can be detected locally, thus enabling on-the-fly LTS reductions. We present an analogous result that might have similar applications, namely, every $\tau$-transition that is sharp $\tau$-confluent is inert for (divergence-unpreserving) sharp bisimilarity.

**Definition 8 (Sharp $\tau$-confluence).** *Let $P = (\Sigma, A, \longrightarrow, p_{init})$ and $T \subseteq \xrightarrow{\tau}$ be a set of internal transitions. $T$ is sharp $\tau$-confluent w.r.t. a set $A_s$ of strong actions if $\tau \notin A_s$ and for all $(p_0, \tau, p_1) \in T$, $a \in A$, and $p_2 \in \Sigma$: (1) $p_0 \xrightarrow{a} p_2$ implies either $p_1 \xrightarrow{a} p_2$ or there exists $p_3$ such that $p_1 \xrightarrow{a} p_3$ and $(p_2, \tau, p_3) \in T$, and (2) if $a \in A_s$ then $p_1 \xrightarrow{a} p_3$ implies either $p_0 \xrightarrow{a} p_3$ or there exists $p_2$ such that $p_1 \xrightarrow{a} p_2$ and $(p_2, \tau, p_3) \in T$. A transition $p_0 \xrightarrow{\tau} p_1$ is sharp $\tau$-confluent w.r.t. $A_s$ if there is a set of transitions $T$ that is sharp $\tau$-confluent w.r.t. $A_s$ and such that $(p_0, \tau, p_1) \in T$.*

The difference between strong $\tau$-confluence and sharp $\tau$-confluence is the addition of condition (2), which can be removed to obtain the very same definition of strong $\tau$-confluence as [28]. Strong $\tau$-confluence thus coincides with sharp $\tau$-confluence w.r.t. the empty set of actions. Sharp $\tau$-confluence not only requires that other transitions of the source state of a confluent transition also exist in the target state, but also that the converse is true for strong actions.

If a transition is sharp $\tau$-confluent w.r.t. $A_s$, then it is also sharp $\tau$-confluent w.r.t. any subset of $A_s$. In particular, sharp $\tau$-confluence is stronger than strong $\tau$-confluence (which is itself stronger than $\tau$-confluence). Theorem 6 formalizes the relationship between sharp $\tau$-confluence and divergence-unpreserving sharp bisimilarity. This result could be lifted to sharp bisimilarity by adding a condition on divergence in the definition of sharp $\tau$-confluence.

**Theorem 6.** *If $\tau \notin A_s$ and $p_0 \xrightarrow{\tau}_P p_1$ is sharp $\tau$-confluent w.r.t. $A_s$, then $p_0$ and $p_1$ are divergence-unpreserving sharp bisimilar w.r.t. $A_s$.*

Theorem 6 illustrates a form of reduction that one can expect using sharp bisimilarity when $\tau \notin A_s$, namely compression of diamonds of sharp $\tau$-confluent transitions, which are usually generated by parallel composition. The strongest form of sharp $\tau$-confluence (which could be called *ultra-strong* $\tau$-confluence) is when all visible actions are strong. In that case, every visible action present in the source state must be also present in the target state, and conversely. The source and target states are then sharp bisimilar w.r.t. the set of visible actions. Yet, it is interesting to note that they are not necessarily strongly bisimilar, sharp bisimilarity w.r.t. all visible actions being weaker than strong bisimilarity.

There exist weaker forms of $\tau$-confluence [27, 50], which accept that choices between $\tau$-confluent and other transitions are closed by arbitrary sequences of $\tau$-confluent transitions rather than sequences of length 0 or 1. It could be interesting to investigate how the definition of sharp $\tau$-confluence could also be weakened, while preserving inertness for sharp bisimilarity.

## 5 LTS Reduction

The interest of sharp bisimilarity in the context of compositional verification is the ability to replace components by smaller but still equivalent ones, as allowed by the congruence property. To do so, we need a procedure that enables such a reduction. This is what we address in this section.

A procedure to reduce an LTS $P$ for sharp bisimilarity is proposed as follows: (1) Build $P'$, consisting of $P$ in which all $\tau$-transitions that immediately precede a transition labelled by a strong action (or all $\tau$-transitions if $\tau$ is itself a strong action) are renamed into a special visible action $\kappa \in \mathcal{A} \setminus A_P$; (2) Minimize $P'$ for divbranching bisimilarity; (3) Hide in the resulting LTS all occurrences of $\kappa$. The renaming of $\tau$-transitions into $\kappa$ allows them to be considered temporarily as visible transitions, so that they are not eliminated by divbranching minimization.[8] This algorithm is now defined formally.

**Definition 9.** *Let $P$ be an LTS and $A_s$ be a set of strong actions. Let $\kappa \in \mathcal{A} \setminus A_P$ be a special visible action. We write $red_{A_s}(P)$ for the reduction of $P$ defined as the LTS "**hide** $\kappa$ **in** $min_{dbr}(P')$", where $P' = (\Sigma_P, A_P \cup \{\kappa\}, \longrightarrow, init(P))$ and $\longrightarrow$ is defined as follows:*

$$\longrightarrow \; = \{(p, \kappa, p') \mid p \xrightarrow{a}_P p' \wedge \underline{\kappa}(a, p')\} \cup \{(p, a, p') \mid p \xrightarrow{a}_P p' \wedge \neg \underline{\kappa}(a, p')\}$$
$$\text{where } \underline{\kappa}(a, p') = ((a = \tau) \wedge (\tau \in A_s \vee p' \xrightarrow{A_s}_P))$$

It is clear that $red_{A_s}(P)$ is a reduction, i.e., it cannot have more states and transitions than $P$. Since the complexities of the transformation from $P$ to $P'$ and of hiding $\kappa$ are at worst linear in $|P|_{tr}$, the complexity of the whole algorithm is dominated by divbranching minimization, for which there exists an algorithm[9] of worst-case complexity $\mathcal{O}(m \log n)$, where $m = |P|_{tr}$ and $n = |P|_{st}$ [25].

As regards correctness, Theorem 7 states that $red_{A_s}(P)$ is indeed sharp bisimilar to $P$. Theorem 8 indicates that the reduction coincides with divbranching minimization if the LTS does not contain any strong action, with strong minimization if $\tau$ is a strong action or if the LTS does not contain $\tau$, and that the resulting LTS has a size that lies in between the size of the minimal LTS for divbranching bisimilarity and the size of the minimal LTS for strong bisimilarity.

**Theorem 7.** *For any LTS $P$, we have $P \sim_{\sharp A_s} red_{A_s}(P)$.*

[8] The letter $\kappa$ stands for *keep uncompressed*.

[9] Strictly speaking, the algorithm of [25] implements branching minimization but, as noted by its authors, handling divergences requires only a minor adaptation.

**Theorem 8.** *The following hold for any LTS P: (1) if* $A_P \cap A_s = \emptyset$ *then* $red_{A_s}(P) = min_{dbr}(P)$*, (2) if* $\tau \notin A_P \setminus A_s$ *then* $red_{A_s}(P) = min_{str}(P)$*, and (3)* $|min_{dbr}(P)|_{st} \leq |red_{A_s}(P)|_{st} \leq |min_{str}(P)|_{st} \wedge |min_{dbr}(P)|_{tr} \leq |red_{A_s}(P)|_{tr} \leq |min_{str}(P)|_{tr}$.

Although sharp reduction is effective in practice, as will be illustrated in the next section, it may fail to compress $\tau$-transitions that are inert for sharp bisimilarity, as show the following examples.

*Example 5.* Consider the LTS of Figure 2(a) (page 9). Its reduction using the above algorithm consists of the three steps depicted below:

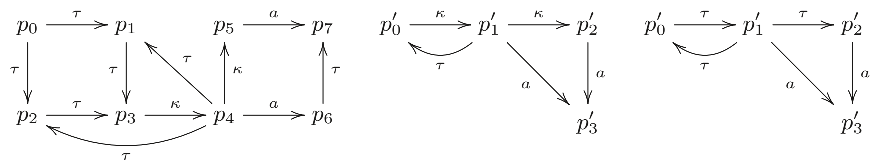

1. $\tau$-to-$\kappa$ renaming 2. Divbranching min. 3. $\kappa$-hiding

The reduced LTS (obtained at step 3) has one more state and two more transitions than the minimal LTS shown in Figure 2(b). Even though all visible actions are strong, our reduction compresses more than strong bisimilarity (recall that the minimal LTS for strong bisimilarity has 7 states and 8 transitions). In general, our reduction reduces more than strong bisimilarity[10] as soon as $\tau \notin A_s$ (which is the case for most formulas in practice).

*Example 6.* In Figure 1 (page 8), if $a \in A_s$ then $red_{A_s}(P_1) = P'_1$, $red_{A_s}(P_2) = P'_2$, and $red_{A_s}(P_6) = P'_6$, i.e., reduction yields the minimal LTS. Yet, $red_{A_s}(P_3) = P_3 \neq P'_3$, i.e., the sharp $\tau$-confluent transition $p_0 \xrightarrow{\tau}_{P_3} p_2$ is not compressed. Similarly, $P_4$, $P_5$, and $P_7$ are not minimized using $red_{A_s}$.

Devising a minimization algorithm for sharp bisimilarity is left for future work. It could combine elements of existing partition-refinement algorithms for strong and divbranching minimizations, but the following difficulty must be taken into account (basic knowledge about partition-refinement is assumed):

– A sequence of $\tau$-transitions is inert w.r.t. the current state partition if both its source, target, and intermediate states are in the same block. To refine a partition for sharp bisimilarity, one must be able to compute efficiently the set of non-inert transitions labelled by weak actions and reachable after an arbitrary sequence of inert transitions. The potential presence of inert cycles has to be considered carefully to avoid useless computations.

[10] The result of reduction is necessarily strong-bisimulation minimal, because if a transition $p \xrightarrow{\tau} p'$ is renamed into $\kappa$, then it is also the case of a $\tau$-transition in every state bisimilar to $p$, which remains bisimilar after the renaming. In addition, the subsequent divbranching minimization step necessarily merges strongly bisimilar states.

– In the case of divbranching bisimilarity, every $\tau$-cycle is inert and can thus be compressed into a single state. This is usually done initially, using the Tarjan algorithm for finding strongly connected components, whose complexity is linear in the LTS size. This guarantees the absence of inert cycles (except self $\tau$-loops) all along the subsequent partition-refinement steps. However, $\tau$-cycles are not necessarily inert for sharp bisimilarity, as illustrated by LTS $P_7'$ in Figure 1 (page 8). Therefore, $\tau$-cycles cannot be compressed initially. Instead, a cycle inert w.r.t. the current partition may be split into several sub-blocks during a refinement step. To know whether the sub-blocks still contain inert cycles, the Tarjan algorithm may have to be applied again.

Although $red_{A_s}$ is not a minimization, we will see that it performs very well when used in a compositional setting. The reason is that (1) only a few of the system actions are strong, which limits the number of $\tau$-transitions renamed to $\kappa$, and (2) sharp $\tau$-confluent transitions most often originate from the interleaving of $\tau$-transitions that are inert in the components of parallel composition. The above reduction algorithm removes most inert transitions in individual (sequential) LTS, thus limiting the number of sharp $\tau$-confluent transitions in intermediate LTS. Still, better reductions can be expected with a full minimization algorithm, which will compress all $\tau$-transitions that are inert for sharp bisimilarity.

## 6 Experimentation

We experimented sharp reduction on the examples presented in [35] (consisting of formulas containing both weak and strong modalities), namely the TFTP (Trivial File Transfer Protocol) and the CTL verification problems on parallel systems of the RERS 2018 challenge. For lack of space, see [35] for more details about these case studies. In both cases, we composed parallel processes in the same order as we did using the combined bisimulations approach, but using sharp bisimilarity instead of strong or divbranching bisimilarity to reduce processes. Experiments were done on a 3GHz/12GB RAM/8-core Intel Xeon computer running Linux, using the specification languages and 32-bit versions of tools provided in the CADP toolbox version 2019-d “Pisa” [19].

The results are given in Figures 3 (TFTP) and 4 (RERS 2018), both in terms of the size of the largest intermediate LTS, the size of the final LTS (LTS obtained after the last reduction step, on which the formula is checked), memory consumption, and time. Each subfigure contains three curves corresponding to the mono-bisimulation approach (using strong bisimulation to reduce all LTS), the combined bisimulations approach, and the sharp bisimulation approach. The former two curves are made from data that were already presented in [35]. Note that the vertical axis of all subfigures is on a logarithmic scale. In the RERS 2018 case, the mono-bisimulation approach gives results only for experiments 101#22 and 101#23, all other experiments failing due to state space explosion.[11]

[11] E.g., smart mono-bisimulation fails on problem 103#23 after generating an intermediate LTS with more than 4.5 billion states and 36 billion transitions (instead of $50,301$ states and $334,530$ transitions using sharp bisimulation) using Grid’5000 [6].

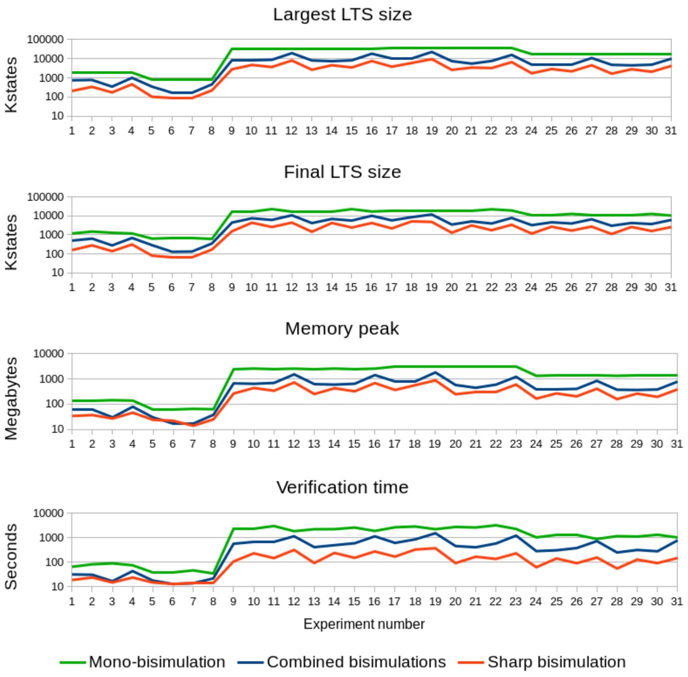

**Fig. 3.** Experimental results of the TFTP case-study

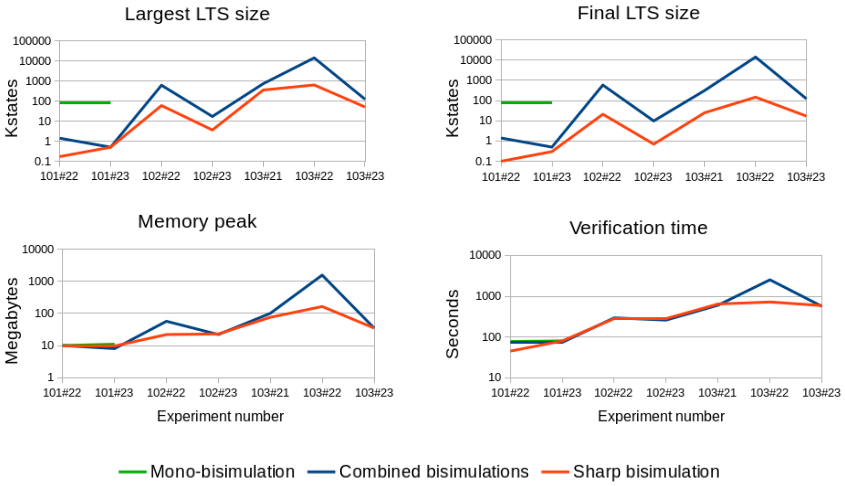

**Fig. 4.** Experimental results of the RERS 2018 case-study

These results show that sharp bisimilarity incurs much more LTS reduction than the combined bisimulations approach, by a factor close to the one obtained when switching from the mono-bisimulation approach to the combined bisimulations approach. However, in the case of the RERS 2018 examples, this gain on LTS size does not always apply to time and/or memory consumption in the same proportions, except for experiment 103#22. This suggests that our implementation of minimization could be improved.

These experiments were conducted after closing of the RERS 2018 challenge. Encouraged by the good results obtained with these two approaches, we participated to the 2019 edition[12], where 180 CTL problems were proposed instead of 9 in 2018. The models on which the properties had to be verified have from 8 to 70 parallel processes and from 29 to 234 actions. Although the models had been given in a wealth of different input formats (communicating automata, Petri nets in PNML format with NUPN information [16], and Promela) suitable for a large number of model checking tools, no other team than ours participated to the parallel challenges. This is a significant difference with 2018, when the challenge was easier, allowing three teams (with different tools) to participate.

We applied smart sharp reduction to these problems, using a prototype program that extracts strong actions automatically from (a restricted set of) CTL formulas used in the competition.[13] This allowed the 180 properties to be checked automatically in less than 2.5 hours (CPU time), and using about 200 MB of RAM only, whereas using strong reduction failed on most of the largest problems. The largest intermediate graph obtained for the whole set of problems has 3364 states. All results were correct and we won all gold medals[14] in this category.[15] Details are available in the Zenodo archive mentioned in the introduction.

## 7 Related Work

The paper [48] defines on doubly-labelled transition systems (mix between Kripke structure and LTS) a family of bisimilarity relations derived from divbranching bisimilarity, parameterized by a natural number $n$, which preserves CTL* formulas whose nesting of next operators is smaller or equal to $n$. Similar to our work, they show that this family of relations (which is distinct from sharp bisimilarity in that there is no distinction between weak and strong actions) fills the gap between strong and divbranching bisimilarities. They apply their bisimilarity relation to slicing rather than compositional verification.

The paper [2] proposes that, if the formula contains only so-called *selective* modalities, of the form $\langle(\neg\alpha_1)^*.\alpha_2\rangle\,\varphi_0$, then all actions but those satisfying

[12] http://rers-challenge.org/2019

[13] The paper [35] presents identities that were used to extract such strong actions.

[14] A RERS gold medal is not a ranking but an achievement, not weakened by the low number of competitors. We also won all gold medals in the “verification of LTL properties on parallel systems” category, using an adaptation of this approach.

[15] http://cadp.inria.fr/news12.html

$\alpha_1$ or $\alpha_2$ can be hidden, and the resulting system can be reduced for $\tau^*.a$-equivalence [14]. Yet, there exist formulas whose strong modalities $\langle\alpha\rangle\,\varphi_0$ cannot translate into anything but the selective modality $\langle(\neg\mathsf{true})^*.\alpha\rangle\,\varphi$, meaning that no action at all can be hidden. In this case, $\tau^*.a$ equivalence coincides with strong bisimilarity and thus incurs much less reduction than sharp bisimilarity. Moreover, it is well-known that $\tau^*.a$-equivalence is not a congruence for parallel composition [7], which makes it unsuitable to compositional verification, even to check formulas that contain weak modalities only.

The adequacy of $L_\mu^{dbr}$ with divbranching bisimilarity is shown in [37]. This paper also claims that ACTL\X is as expressive as $L_\mu^{dbr}$ and thus also adequate with divbranching bisimilarity, but a small mistake in the proof had the authors omit that the $L_\mu^{dbr}$ formula $\langle\tau\rangle\,@$ cannot actually be expressed in ACTL\X. It remains true that ACTL\X is preserved by divbranching bisimilarity.

In [13], it is shown that ACTL\X is adequate with divergence sensitive branching bisimilarity. This bisimilarity relation is equivalent to divbranching bisimilarity [21–23] only in the case of deadlock-free LTS, but it differs in the presence of deadlock states since it does not distinguish a deadlock state from a self $\tau$-loop (which can instead be recognized in $L_\mu^{dbr}$ with the $\langle\tau\rangle\,@$ formula).

## 8 Conclusion

This work enhances the reductions that can be obtained by combining compositional LTS construction with an analysis of the temporal logic formula to be verified. In particular, known results about strong and divbranching bisimilarities have been combined into a new family of relations called sharp bisimilarities, which inherit all nice properties of their ancestors and refine the state of the art in compositional verification.

This new approach is promising. Yet, to be both usable by non-experts and fully efficient, at least two components are still missing: (1) The sets of strong actions, which are a key ingredient in the success of this approach, still have to be computed either using pencil and paper or using tools dedicated to restricted logics; automating their computation in the case of arbitrary $L_\mu$ formulas is not easy, but likely feasible, opening the way to a new research track; finding a minimal set of strong actions automatically is challenging, and since it is not unique, even more challenging is the quest for the set that will incur the best reductions. (2) Efficient algorithms are needed to minimize LTS for sharp bisimilarity; they could probably be obtained by adapting the known algorithms for strong and divbranching minimizations (at least using some kind of signature-based partition refinement algorithm in the style of Blom *et al.* [3–5] in a first step), but this remains to be done.

*Acknowledgements.* The authors thank Hubert Garavel, who triggered our collaboration between Grenoble and Pisa, and Wendelin Serwe for his comments on earlier versions of this paper. They also thank the anonymous referees for the pertinence of their comments, which allowed significant improvements of this paper.
